# Nitrate Regulates Maize Root Transcriptome through Nitric Oxide Dependent and Independent Mechanisms

**DOI:** 10.3390/ijms22179527

**Published:** 2021-09-02

**Authors:** Laura Ravazzolo, Sara Trevisan, Silvia Iori, Cristian Forestan, Mario Malagoli, Silvia Quaggiotti

**Affiliations:** 1 Department of Agronomy, Food, Natural Resources, Animals and Environment (DAFNAE), University of Padua, Viale dell’Università 16, 35020 Legnaro, Italy; laura.ravazzolo@unipd.it (L.R.); sara.trevisan@unipd.it (S.T.); mario.malagoli@unipd.it (M.M.); 2 Department of Comparative Biomedicine and Food Science (BCA), University of Padua, Viale dell’Università 16, 35020 Legnaro, Italy; silvia.iori@unipd.it; 3 Department of Agricultural and Food Sciences (DISTAL), University of Bologna, Viale Fanin 44, 40127 Bologna, Italy; cristian.forestan@unibo.it

**Keywords:** maize, nitrate, nitric oxide, root, signaling, transcriptome

## Abstract

Maize root responds to nitrate by modulating its development through the coordinated action of many interacting players. Nitric oxide is produced in primary root early after the nitrate provision, thus inducing root elongation. In this study, RNA sequencing was applied to discover the main molecular signatures distinguishing the response of maize root to nitrate according to their dependency on, or independency of, nitric oxide, thus discriminating the signaling pathways regulated by nitrate through nitric oxide from those regulated by nitrate itself of by further downstream factors. A set of subsequent detailed functional annotation tools (Gene Ontology enrichment, MapMan, KEGG reconstruction pathway, transcription factors detection) were used to gain further information and the lateral root density was measured both in the presence of nitrate and in the presence of nitrate plus cPTIO, a specific NO scavenger, and compared to that observed for N-depleted roots. Our results led us to identify six clusters of transcripts according to their responsiveness to nitric oxide and to their regulation by nitrate provision. In general, shared and specific features for the six clusters were identified, allowing us to determine the overall root response to nitrate according to its dependency on nitric oxide.

## 1. Introduction

Nitrogen (N) impacts crop production and its availability in soil strongly depends on the use of N fertilizers [1]. However, the average rate of N recovery of crops is low, determining rises in the cost of agricultural production and environmental degradation [2,3].

Nevertheless, even in fertilized arable soil, a large portion of N available for crops relies on the slow mineralization of organic N [4,5], generating the need for specific strategies for exploring more soil space with a larger root system that is positively correlated with higher N use efficiency in crops [6].

In aerated soils, ammonium is predominantly transformed into nitrate, which shows a concentration 10–1000 times higher than that of ammonium [7], thus representing the prevalent form through which crops uptake N [8].

The improvement of root traits has been acknowledged as a crucial element for the second green revolution [9] and the achievement of new N-efficient cultivars will help sustainable agriculture development [10]. The knowledge of the physiological and molecular mechanisms underlying the desirable traits is a necessary requirement to develop reliable breeding strategies to improve N use efficiency, also considering the high degree of genotypic variation among species and cultivar observed for root architecture [11,12,13,14]. Nitrate regulation of root development is complex, and it involves several signaling pathways which have been only partially decoded. Their understanding will be a key factor to select genotypes more efficient in N use.

Previous research in maize [15,16,17,18] evidenced the role of nitric oxide (NO) as a signaling molecule involved in the early induction of primary root elongation in response to nitrate, probably controlling the balance between cell proliferation and cell elongation [19]. NO is a small gaseous molecule and in plants, it can be produced by nitric oxide synthase (NOS), nitrate reductase (NR), xanthine oxidase (XOS), and non-enzymatic reactions [20]. It acts as a signal regulating adventitious root formation [21], lateral root development [22], root hair formation [23], and primary root elongation [15,24,25,26].

In maize, NO is rapidly and transiently produced in a specific portion of primary root located between the meristem and the elongation zone, namely the transition zone, by nitrate reductase (NR) soon after the provision of nitrate to N-deprived roots, thus inducing primary root to elongate [15].

This NO burst seems to affect auxin polar transport and localization, possibly turning off the biosynthesis and transport of strigolactones (SLs) [27]. Accordingly, Sun et al. [28] showed that in rice, NO produced by the NR pathway under NO_3_^−^ supply induces seminal root elongation by regulating auxin transport. In the same species, NO produced by the NR increases N uptake capacity by regulating lateral root formation and inorganic N uptake [22], supporting the role of NO as a crucial signal for the root adaptation to nitrate availability, at least in maize and rice.

Furthermore, Ravazzolo et al. [29] demonstrated that N-deficiency strongly induces SL exudation by maize roots and that the supply of nitrate rapidly switches off this process. More recently, it has been also suggested that nitrate induces lateral root development at least in part through auxin and that this mechanism depends on the upstream inhibition of SL production observed after nitrate supply [30]. Previous results by Manoli et al. [27] seem to suggest that NO production, being an early fine-tuned signature of nitrate perception, might induce SL inhibition and the concomitant auxin action, thus lastly regulating the lateral root growth.

In accordance with these results, nitrate availability would seem to be perceived by maize root and translated in a physiological and developmental response thanks to a complex interplay among NO, SLs, and auxin. However, the precise contribution and the reciprocal regulatory role of these molecules needs to be further understood.

cPTIO (2-4-carboxyphenyl-4,4,5,5-tetramethylimidazoline-1-oxyl-3-oxide) is a highly specific NO scavenger [31] and it is a useful tool to dissect the effects depending on NO from those which are NO-independent [32]. Due to its complex chemical properties, the scavenging abilities of cPTIO seem to be significantly reduced in a time-dependent manner and it is not always able to completely scavenge NO, especially for treatments inducing a gradual and continuous production of NO [33]. However, in our case, NO biosynthesis has been demonstrated to be rapidly and transiently triggered by nitrate provision to N-depleted maize roots and the cPTIO provision was demonstrated to be able to significantly reduce the amount of NO in the primary root apex [15].

The present work was aimed at studying the impact of cPTIO provision on the expression of genes modulated by nitrate. In order to dissect the molecular response to nitrate according to its dependency or independency on nitric oxide, an RNA-sequencing comparative analysis was performed. The expression of transcripts responsive to nitrate was then evaluated also in function of their responsiveness to cPTIO, enabling discrimination between NO-dependent and NO-independent mechanisms of regulation. A subsequent detailed functional characterization of DEGs by means of Gene Ontology (GO) and MapMan enrichment, transcription factors detection, and KEGG metabolic pathways investigation led us to evidence common and specific features for six different clusters of genes and to hypothesize their participation in specific signaling and metabolic pathways in the maize root response to nitrate fluctuations. Finally, considering the relevance of nitrate signal in the root development, the lateral root density was measured both in the presence of nitrate and in the presence of nitrate plus cPTIO and compared to that observed for N-depleted roots.

## 2. Results

### 2.1. Reads Processing and Differential Expression Analysis

Transcriptomic profiles of maize root apexes grown in N-deprivation (−N) compared to those grown with nitrate supply (+NO_3_^−^) were analyzed and discussed in a previous work [34]. Here, we further investigated the transcriptional regulation of root response to NO_3_^−^ provision, using cPTIO as nitric oxide (NO) scavenger to block NO signaling in roots. This allows us to distinguish, among NO_3_^−^ responsive genes, those showing a regulation NO-dependent from NO-independent. After trimming and filtering, high-quality reads were mapped on the maize B73 v4 reference genome (Jiao et al. 2017), then used for estimation of gene expression levels in the three different conditions (expressed as Reads Per Kb per Million, RPKM; Table S1) and for pairwise differential expression analysis. Genes showing a fold change >2 (corresponding to a log2 fold change ratio >|1|) and a false discovery rate (FDR)-adjusted *p*-value ≤ 0.05 were considered as differentially expressed genes (DEGs).

**Figure 1:**
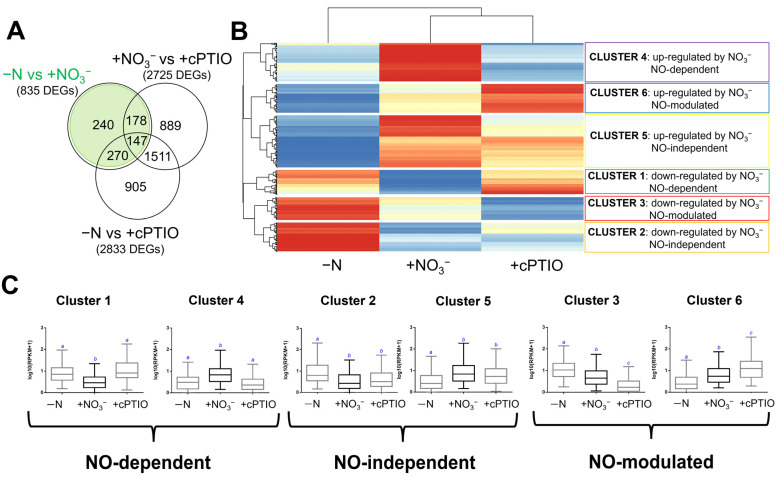
Venn and clustering analysis of genes differentially expressed in +NO_3_^−^ and +NO_3_^−^ + cPTIO treatments with respect to the control (−N) in maize root tissues. (**A**) Venn diagram showing the numerical comparison of all significant (log2 FC > |1|; FDR ≤ 0.05) differential expressed genes (DEGs) following −N, +NO_3_^−^, and +NO_3_^−^ + cPTIO treatments. (**B**) Clustered heat map of expression changes in pairwise comparison between −N (control), +NO_3_^−^ and + cPTIO treated roots, for the 835 shared DEGs. DEGs normalized z-scaled expression values are reported in a blue to red color scale (blue: lower RPKM values, red: higher RPKM values). (**C**) Boxplot of normalized expression values presented as log10 transformed RPKM for the genes included in each cluster; blue letters indicate Mann–Whitney statistics test with *p* < 0.01. The analysis reveals 6 clusters that could be described by the expression behaviors in response to NO_3_^−^ provision and NO-(un)responsiveness: Cluster 1 (NO-dependent and down-regulated by NO_3_^−^), cluster 2 (NO-independent and down-regulated by NO_3_^−^), cluster 3 (NO-modulated and down-regulated by NO_3_^−^), cluster 4 (NO-dependent and up-regulated by NO_3_^−^), cluster 5 (NO-independent and up-regulated by NO_3_^−^), and cluster 6 (NO-modulated and up-regulated by NO_3_^−^). RPKM: Reads Per Kb per Million; FC: Fold change.

Pairwise comparison between nitrate-supplied plants (+NO_3_^−^) and NO-scavenged ones (+NO_3_^−^ + cPTIO) resulted in 2725 DEGs significantly responsive to cPTIO treatment, while pairwise comparison between nitrate-deficient plants (−N) and NO-scavenged ones (+NO_3_^−^ + cPTIO) resulted in 2833 DEGs. DEGs identified in these two pairwise comparisons were then compared to the 835 DEGs previously found [34] as directly regulated by the nitrate provision (+NO_3_^−^), if compared to N-starvation (−N; Figure 1A), analyzing how they respond to the other treatments (−N; +NO_3_^−^ + cPTIO). The 3305 DEGs that seemed responsive only to cPTIO, but not directly to nitrate application, were not further considered in this study (Figure 1A).

To better dissect the NO-dependent from the NO-independent components of gene expression regulation in response to nitrate, a hierarchical clustering of the 835 DEGs was then performed (Figure 1B). DEGs were grouped into six clusters according to their responsiveness to NO_3_^−^ provision and to their regulation by NO (as resulted from cPTIO application), as displayed in Figure 1B,C. Accordingly, 269 DEGs showed a marked NO-dependency of NO_3_^−^-induced down- or up-regulation and were grouped in clusters 1 and 4, respectively, while 347 DEGs resulted regulated by nitrate regardless of NO production and were grouped in clusters 2 and 5, respectively (Table S2). Heat map and box plots of DEG expression values clearly showed that transcriptional changes induced in response to NO_3_^−^ application were completely impaired by the NO-scavenger cPTIO for NO-dependent genes. On the contrary, cPTIO application did not affect the NO_3_^−^-induced transcriptional modulation in the case of NO-independent DEGs.

In addition to NO-dependent and independent DEGs, two additional gene clusters (3 and 6) were detected, including 219 DEGs in which the effect of the nitrate provision is further amplified by the NO scavenger application. Cluster 3, which includes 97 DEGs down-regulated by nitrate provision, and cluster 6, with 122 DEGs up-regulated by nitrate supply, were defined as NO-modulated groups. Even if NO is not necessary for the NO_3_^−^-transcriptional regulation of these genes, NO scavenging resulted in a magnified NO_3_^−^-induced up- or down-regulation, indicating that their transcriptional regulation by nitrate does not depend on NO production, even thought they could be regulated by NO regardless of nitrate. Therefore, in this context, they should be fully considered as nitrate regulated in an NO-independent way.

In detail, among the 269 DEGs dependent on NO, 104 DEGs resulted down-regulated by nitrate supply (cluster 1) and 165 up-regulated by nitrate supply (cluster 4), while among the 374 DEGs unresponsive to NO, 124 DEGs were down-regulated in response to nitrate (cluster 2) and 223 up-regulated by nitrate provision (cluster 5). As shown in Figure 1C, NO-dependent clusters 1 and 4 include transcripts in which cPTIO application restores expression levels comparable to those observed in −N. On the contrary, genes belonging to NO-independent clusters 2 and 5 displayed a similar trend of expression in response to NO_3_^−^ and upon cPTIO application. Finally, NO-modulated clusters 3 and 6 comprise transcripts which in response to cPTIO showed expression profiles with a greater magnitude with respect to NO_3_^−^/−N. Accordingly, when nitrate supply induced a down-regulation, the cPTIO provision replicated this negative regulation but to a greater extent (cluster 3, Figure 1C). Similarly, when nitrate provision induced an up-regulation, the cPTIO treatment imitated this induction but to a greater magnitude (cluster 6, Figure 1C).

### 2.2. Annotation and Classification of Clustered DEGs into GO Functional Categories

**Figure 2:**
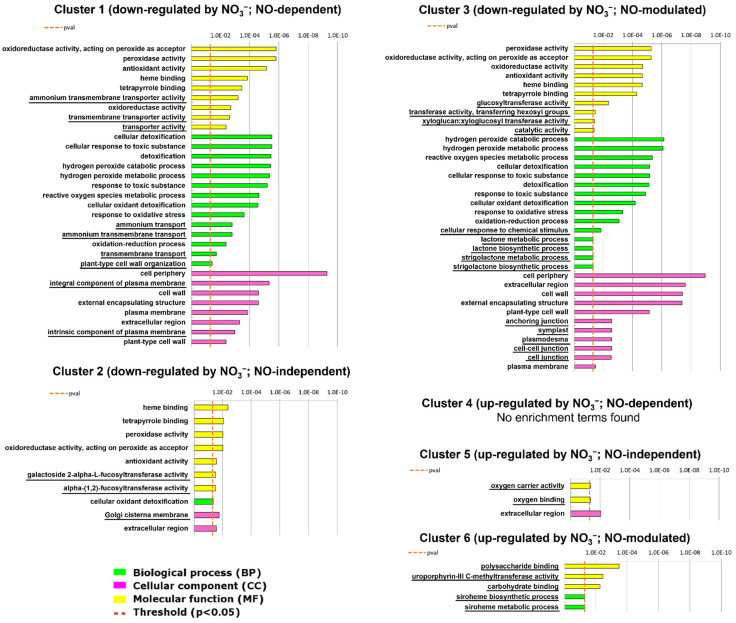
Enrichment analysis of DEGs clustered in 6 groups using gProfiler. The figure shows the GO categories overrepresented among genes down-regulated by nitrate provision and NO-dependent (cluster 1), down-regulated by nitrate and NO-independent (cluster 2), down-regulated by nitrate and NO-modulated (cluster 3), up-regulated by nitrate provision and NO-independent (cluster 5), or up-regulated by nitrate and NO-modulated (cluster 6). The specific and unique terms for each group are underlined. No enriched terms were found among DEGs up-regulated by nitrate provision and NO-dependent (cluster 4).

Gene Ontology (GO) provides a controlled vocabulary for describing biological processes (BP), molecular functions (MF), and cellular components (CC). A GO enrichment analysis was performed to functionally characterize the DEGs included in the six previously identified groups, and the resulting enriched GO terms were further distinguished in shared and unique terms (Figure 2; Table S3).

In Figure 2, GO terms that have been uniquely identified in each group are underlined, highlighting many specific components for the NO-responsiveness and NO-unresponsiveness transcriptional response to nitrate. DEGs belonging to cluster 1 (NO_3_^−^ down-regulated, NO-dependent) were specifically enriched for GO terms related to transmembrane transport, such as ammonium transport, while those NO-independent but still down-regulated by nitrate provision (cluster 2) showed a specific enrichment in GO terms related to the cell wall through a fucosyltransferase activity and Golgi cisterna membrane. Finally, genes included in cluster 3 showed a specific overrepresentation of terms related to catabolic processes, SL biosynthetic and metabolic processes, and cell junction. Conversely, peroxidase and antioxidant activity, cellular detoxification, reactive oxygen species metabolic process and cell wall localization related GO terms resulted concurrently enriched among the DEGs of clusters 1, 2, and 3.

On the other hand, transcripts NO-independent and up-regulated by nitrate (cluster 5) were enriched in terms related to oxygen carrier activity and oxygen binding, while those up-regulated by nitrate and NO-modulated (cluster 6) showed a specific enrichment in terms related to polysaccharide and carbohydrate binding, and siroheme metabolic processes which are involved in the reduction of nitrate or sulphate. No shared GO terms among cluster 5 and 6 were found and no enriched GO terms were detected among genes up-regulated by nitrate and NO-responsive (cluster 4).

### 2.3. MapMan Functional Analysis

**Figure 3:**
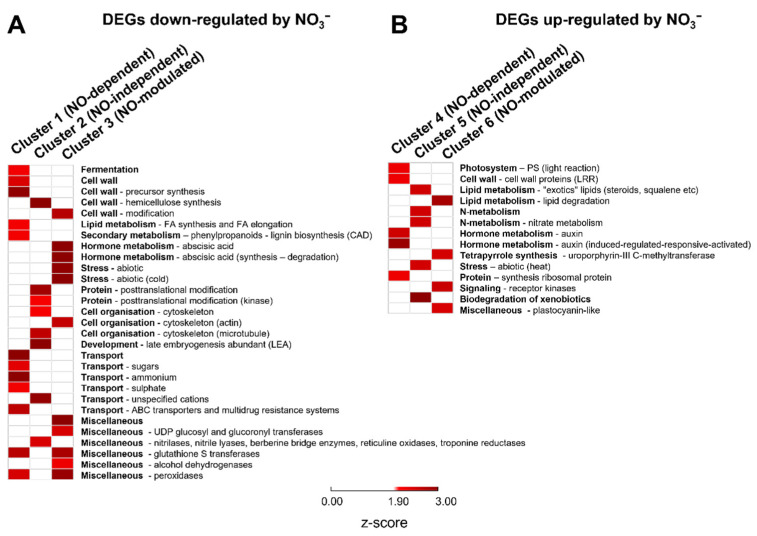
MapMan functional categories enrichment of DEGs identified in the six groups ((**A**) down-regulated by nitrate provision; (**B**) up-regulated by nitrate provision) using the PageMan tool with Fisher’s test. PageMan automatically converted the *p*-values to their corresponding z-scores (e.g., 1.96 for a *p*-value of 0.05). In the map, white boxes represent no enrichment, while different magnitudes of red show increasing significance of the enriched terms.

Functional analyses of DEGs were performed by means of MapMan over-representation analysis. The MapMan ontology comprises a set of 27 tree-structured bins, describing the central metabolism, many regulatory processes such as transcription factors and signaling pathways, as well as biotic and abiotic stress responses. Many MapMan functional categories were found to be enriched among DEGs down-regulated upon nitrate supply, as shown in Figure 3A. For genes belonging to cluster 1 (down-regulated by nitrate and NO-dependent), functional bins related to lipid metabolism through fatty acids synthesis and elongation, transmembrane transport of sugars, ammonium, and sulphate were found. Cluster 2 (down-regulated by nitrate and NO-independent) displayed abundancy of transcripts related to protein post-translational modification, cell organization through the cytoskeleton, and late embryogenesis. Cluster 3 (transcripts modulated by NO, but down-regulated by nitrate independently from NO) showed a significant enrichment in terms related to the abscisic acid metabolism, to the abiotic stress (i.e., cold), and to alcohol dehydrogenases. Finally, shared enriched terms among the DEGs down-regulated by nitrate regardless of the NO actions interested cell wall-related genes, glutathione S transferases, and peroxidases.

Regarding clusters including genes up-regulated after nitrate provision (Figure 3B), fewer MapMan functional categories were found to be enriched, particularly auxin-related bins for cluster 4, N-metabolism, abiotic stress (i.e., heat), and biodegradation of xenobiotics bins for cluster 5, and lipid degradation and tetrapyrrole synthesis bins for cluster 6.

### 2.4. KEGG Analysis

**Figure 4:**
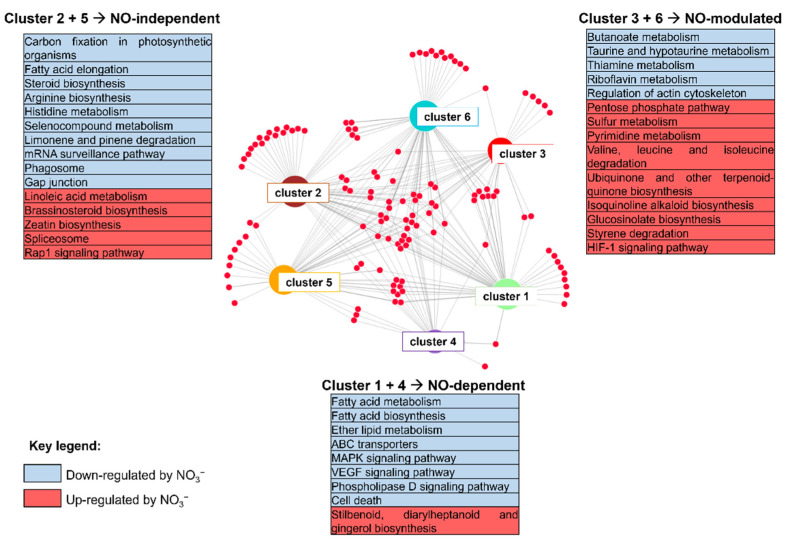
Pathway assignment based on KEGG mapper reconstruction of DEGs specific among the six clusters. Cluster 1, DEGs down-regulated by nitrate provision and NO-dependent; cluster 2, DEGs down-regulated by nitrate and NO-independent; cluster 3, DEGs down-regulated by nitrate and NO-modulated; cluster 4, DEGs up-regulated by nitrate provision and NO-dependent; cluster 5, genes up-regulated by nitrate provision and NO-independent; cluster 6, DEGs up-regulated by nitrate and NO-modulated.

A KEGG mapper pathways reconstruction was used to show pathways related to the six clusters (Figure 4; Table S4). While the multiple annotations in GO terms can lead to a strong redundancy, the KEGG Orthology (KO) database used a hierarchical structure of protein function terms. In addition, KEGG Mapper can be used as a collection of KEGG mapping tools for linking molecular objects such as genes, proteins, and metabolites to higher-level objects, such as pathways.

DEGs were assigned to 55 pathways for cluster 1, 57 pathways for cluster 2, 33 pathways for cluster 3, 21 pathways for cluster 4, 48 pathways for cluster 5, and 54 pathways for cluster 6. Some of these pathways were specifically reconstructed in each cluster, leading to suppose NO-dependent and NO-independent regulation processes, while other pathways were variably shared among two or more clusters (Figure 4).

Pathways related to fatty acid biosynthesis, membrane transport through ABC-type transporters, cell death, and finally, signal transduction through the MAPK- and phospholipase D were specific for genes down-regulated by nitrate and dependent on NO (cluster 1), while pathways related to stilbenoid, diarylheptanoid, and gingerol biosynthesis were specific for genes up-regulated by nitrate and NO-dependent (cluster 4).

Pathways related to brassinosteroids and zeatin biosynthesis, and RAP1-signaling were specific for transcripts up-regulated by nitrate but unresponsive to NO (cluster 5), while the fatty acid elongation and gap junction, and the metabolism of arginine, histidine, and seleNO-compounds were specific for genes down-regulated by nitrate but unresponsive to NO (cluster 2).

Finally, pathways related to thiamine and riboflavin metabolism and to cell motility through the regulation of actin cytoskeleton were specific for transcripts down-regulated by nitrate and NO-modulated (cluster 3), while pyrimidine metabolism, sulfur metabolism and valine, leucine and isoleucine degradation were specific for those up-regulated by nitrate and modulated by NO (cluster 6).

Xenobiotics biodegradation and metabolism-related pathways (i.e., drug metabolism via cytochrome P450, or other enzymes) were shared among clusters 1, 3, and 6, while phenylpropanoid biosynthesis pathway was shared among all clusters, regardless of NO action (Table S4**)**. Remarkably, pathways related to plant hormonal signaling were shared among all clusters, with the only exception for cluster 1 (genes down-regulated by nitrate and NO-dependent). The KEGG Search&Color Pathway tool allowed us to highlight further peculiarities in terms of hormonal pathways among the five clusters (Figure S1). Ethylene signaling was specific for cluster 5, independent from NO, as well as some components of auxin signaling (*SAUR*) were specific in cluster 2. Nevertheless, other auxin-signaling components, such as *Aux/IAA* and *GH3*, were shared between clusters 3 and 4. Regarding genes related to cytokinin biosynthesis, and the abscisic acid, jasmonic acid, and salicylic acid signaling, they all were included in cluster 6, thus being NO-modulated but up-regulated by nitrate independently from NO. Finally, the brassinosteroids signaling was related to the NO-dependent cluster induced by nitrate (cluster 4).

### 2.5. Analysis of Transcription Factors

**Figure 5:**
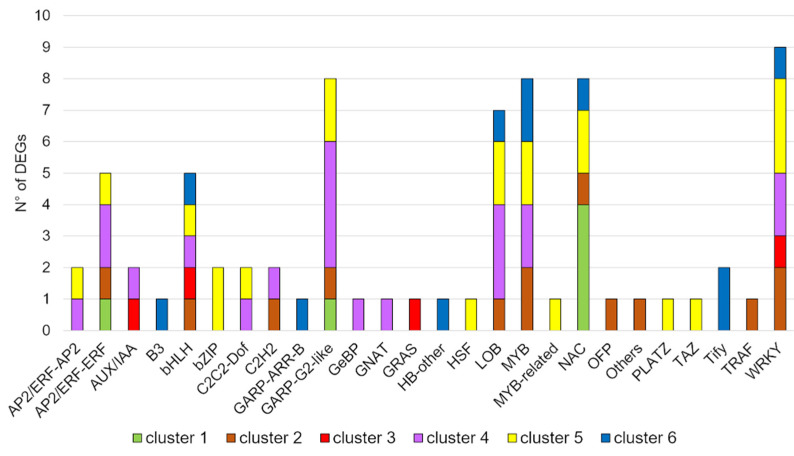
Comparison between transcription factors (TFs) in the six clusters. Cluster 1, DEGs down-regulated by nitrate provision and NO-dependent; cluster 2, DEGs down-regulated by nitrate and NO-independent; cluster 3, DEGs down-regulated by nitrate and NO-modulated; cluster 4, DEGs up-regulated by nitrate provision and NO-dependent; cluster 5, genes up-regulated by nitrate provision and NO-independent; cluster 6, DEGs up-regulated by nitrate and NO-modulated. The number of genes coding for transcription factors were detected using the iTAK database.

The presence of transcription factors (TFs) among DEGs was then evaluated with a targeted search using the iTAK database (Figure 5). A total of 53 TF-encoding genes from 26 TF families were globally identified. Cluster 1 includes 6 of them from 3 TF families, cluster 2 counts 13 of them from 11 TF families, cluster 3 comprises only 4 TF-encoding genes from 4 TF families, cluster 4 includes 20 of them from 12 TF families, cluster 5 exhibits 21 TF-encoding genes from 14 TF families, and cluster 6 encloses 11 TF-encoding genes from 9 TF families. Among the identified TF families, NAC members seemed specific and highly represented in cluster 1, the GARP-G2-like family appeared the most representative for cluster 4, WRKY appeared the most representative TF family for cluster 5 (even it was found also in other clusters), and the Tify family for cluster 6. Nevertheless, bHLH and WRKY appeared equally shared among all clusters, except for cluster 1, while LOB and MYB were common among clusters 5, 4, 2, and 6, suggesting a universal involvement of these four TF families in the maize root response to nitrate regardless of NO action. bZIP, HSF, MYB-related, PLATZ, and TAZ TF-families appeared only in cluster 5, GeBP and GNAT only in cluster 4, OFP and TRAF only in cluster 2, GRAS only in cluster 3, and B3, GARP-ARR-B, and Tify only in cluster 6.

### 2.6. Validation of RNA-seq Data by Real-Time PCR (qRT-PCR)

**Figure 6:**
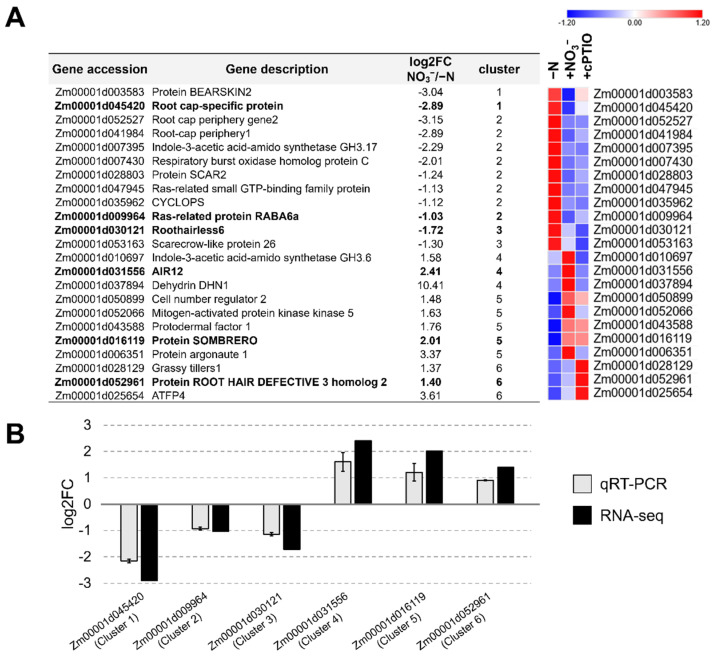
Validation of RNA-seq data by Real-time PCR (qRT-PCR). (**A**) Heat map of expression changes in pairwise comparison between −N (control), +NO_3_^−^ and +cPTIO treated roots, for 23 randomly selected differentially expressed genes (DEGs) among all the six clusters. DEGs normalized z-scaled expression values are reported in a blue to red color scale (blue: lower RPKM values, red: higher RPKM values). In bold are highlighted the six genes used for qRT-PCR. (**B**) Six representative genes were chosen to validate the RNA-seq data by qRT-PCR. The gray bars represent mean values of the log2 fold-change ratio (log2FC) among NO_3_^−^/−N samples obtained from three biological replicates of qRT-PCR, with error bars reporting the standard errors, while the black bars represent the RNA-seq data. RPKM, Reads Per Kb per Million.

Twenty-three genes belonging to all six clusters were randomly selected according to both their transcription profiles and their putative functions, and their expression levels were shown in a heat map figure (Figure 6A). Among them, one gene for each cluster was randomly selected, and their transcription levels were measured in the −N and +NO_3_^−^ samples (Figure 6B). The list of the six selected genes with the primers used for the gene expression analysis are reported in Table S5. The qRT-PCR results confirmed the RNA-seq profiles, even if the entity of their fluctuations varied with respect to RNA-Seq data, probably because of the higher sensitivity of the RNA-seq analysis.

### 2.7. No Impact on Maize Seedlings Growth

**Figure 7:**
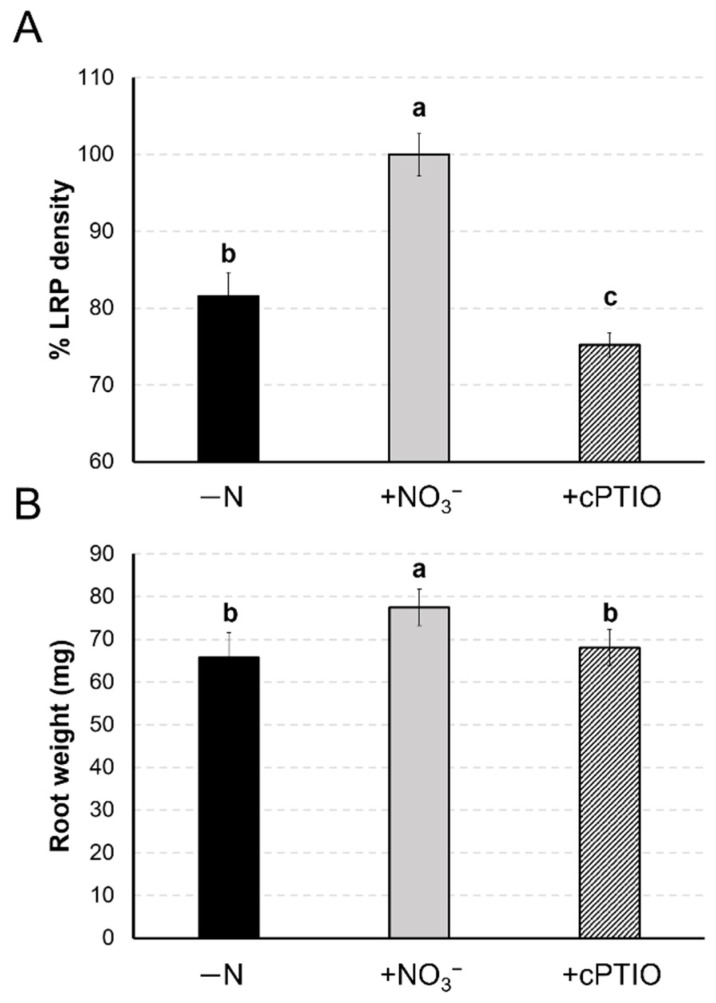
Lateral root density (LRP) and root weight of maize seedlings exposed to NO scavenger. Maize seedlings were grown in an N-depleted solution for 24 h and then transferred for 24 h in a nitrogen depleted solution (−N), or 24 h in a 1 mM nitrate supplied solution (+NO_3_^−^), or 24 h in a 1 mM NO_3_^−^ supplied solution plus 1 mM NO scavenger cPTIO (cPTIO). Hematoxylin staining was used to detect lateral root primordia (LRP). (**A**) Data are expressed as increment of LRP density respect to the +NO_3_^−^ treated roots. Roots of all maize seedlings were separated and individually weighed. (**B**) The average weight of roots was calculated for every biological repetition and then averaged between the three biological repetitions. Results are presented as mean ± SE from three biological replicates for each treatment and an ANOVA statistic test was performed (*n* = 20). Letters above the bars indicate different significance groups (*p* < 0.05).

To dissect the direct effect of NO on the maize seedlings root growth, lateral root density (LRP) and root weights were measured in response to the NO scavenger cPTIO (Figure 7). Nitrate-provided plants always showed a significantly higher number of LRP (+18.4%) compared to plants grown in an N-free solution (−N), as already reported by Ravazzolo et al. [29]. However, when cPTIO was provided together with nitrate, a marked decrease in LRP density (−24.8%) was noticed (Figure 7A). As far as the root biomass accumulation was concerned, a significant increase in their fresh weights was observed in response to NO_3_^−^ (+20%), but the provision of cPTIO restored the −N phenotype (Figure 7B).

## 3. Discussion

Nitrate availability strongly affects root development [35,36,37,38]. Previous works on maize seedlings evidence the role played by nitric oxide (NO) produced by nitrate reductase (NR) in inducing the root apex to elongate soon after nitrate provision [15,16,17]. Further studies also showed that nitric oxide participates in many aspects involved in root development, as summarized by Trevisan et al. [16] and Freschi [39].

Besides NO, strigolactones (SLs) also are supposed to take part to the nitrate regulation of root development in maize, possibly through a combined action with auxin [20,29,30]. However, the exact interplay among NO, SLs, and auxin in the overall process is still far from being fully understood.

Thanks to a combined pharmacological and molecular approach, the participation of NO in the regulation of the maize root response during the first 24 h after nitrate provision was deepened. Our results (Figure 1) showed that 32% of the nitrate-regulated genes were NO-responsive (clusters 1 with genes down-regulated by nitrate provision, and cluster 4 with genes up-regulated by nitrate supply), whilst 42% of them displayed a regulation of their transcription regardless of NO production (clusters 2 with genes down-regulated by nitrate provision, and cluster 5 with genes up-regulated by nitrate supply). Furthermore, 26% of the nitrate-regulated genes, despite being modulated by NO (clusters 3 with genes down-regulated by nitrate provision, and cluster 6 with genes up-regulated by nitrate supply), are supposed to be regulated by nitrate in an NO-independent mode, since cPTIO provision strongly heightened the nitrate-induced gene expression.

The comparison of the main GO categories enriched in the six clusters (Figure 2; Table S3) revealed that the overall root response to nitrate could be decomposed in accordance with its dependency on NO and that specific pathways distinguish this response depending on the downstream effector (NO, nitrate itself or any further molecule belonging to the assimilation pathway).

KEGG ontology and MapMan enrichment provided further information on metabolic and signaling processes featuring the maize root response to nitrate and mediated by NO or dependent on further downstream events. As already showed by GO analysis (Figure 2), the MapMan analysis (Figure 3) and the KEGG Reconstruction (Figure 4) clearly indicates that many processes are specifically regulated in response to NO biosynthesis, while few others seem to mainly depend on further signals.

Transcripts down-regulated by nitrate in an NO-dependent manner (cluster 1) were enriched in terms related to specific transmembrane transport activity, in particular, of ammonium (Figure 2), sugars, and sulphate (Figure 3) and in terms related to phospholipase D- and MAPK signaling (Figure 4). The link between NO- and MAPK- signaling has been already revealed in diverse physiological processes [40], such as adventitious root development in cucumber [41,42] and isoflavone accumulation in soybean [43]. Furthermore, fatty acids biosynthesis/degradation and lipid metabolism pathways were also included in cluster 1 (Figure 4), probably being involved in the regulation of the cell membrane development. Accordingly, the lipid biosynthesis was shown to be significantly altered by N nutrition in Arabidopsis [44,45] and in *Camellia sinensis* L. [46]. Moreover, cluster 1 is the only cluster with no DEGs encoding hormone-related targets (Figure S1), but it is enriched in NAC-type transcription factors (Figure 5), having functions in nutrient distribution, cell wall biosynthesis, and abiotic and biotic stress responses [47].

The cluster of transcripts up-regulated by nitrate in an NO-dependent manner (cluster 4) was particularly enriched in TF-encoding genes (Figure 5), such as the GARP-G2-like family, which is usually associated with chloroplast development, but also with abiotic stress resistance [48], and LOB family, which is related to root development [49]. In addition, a correlation with auxin signaling through Aux/IAA transcriptional regulators and *GH3* auxin-responsive gene, and with brassinosteroids response through the induction of *TCH4, a* xyloglucan endotransglucosylase/hydrolase encoding gene [50] was clearly evidenced for this cluster (Figure S1).

Genes regulated by nitrate provision and defined as NO-modulated (clusters 3 and 6) should be considered as NO-unresponsive, since the cPTIO provision do not reverse the nitrate effect on their transcriptional regulation. Among them, cluster 3 (down-regulated by nitrate) includes the regulation of actin cytoskeleton by the *actin depolymerizing factor 13* (*ADF13*, Zm00001d017516) and *actin depolymerizing factor 7* (*ADF7*, Zm00001d051388) as a typical feature (Figure 4). ADF is a small class of actin-binding proteins that regulates the dynamics of actin in cells. Recently, Huang et al. [51] showed that maize *ADF13* is significantly upregulated upon abiotic stress such as drought stress, while *ADF7* appeared involved in cold resistance. Actually, cluster 3 was enriched for the abiotic stress bin regarding cold (Figure 3). It also included two genes encoding components of strigolactone biosynthesis and metabolism (Zm00001d002736 encoding the ‘*carotenoid cleavage dioxygenase 7′* and Zm00001d043442 encoding the ‘*carotenoid cleavage dioxygenase 8′*; Figure 2; Table S3), suggesting that the involvement of SL in response to N, at least in our conditions, takes place independently from NO production. Nevertheless, in sunflower, it has been demonstrated that NO regulates the lateral root development through a reversible inhibition of CCD activity [52], and in rice, Sun et al. [53] suggested that NO targeted D53, a repressor of SLs, thanks to a proteasome-mediated degradation, as also confirmed by Zhou et al. [54] and Jiang et al. [55].

As far as cluster 6 is concerned, it was characterized by polysaccharide binding terms (Figure 2), together with some terms involved in defense response-related hormone metabolism, such as jasmonic acid (JA), salicylates (SA), and abscisic acid (ABA) (Figure S1). In addition, it was significantly enriched in Tify-type transcription factors (Figure 5). Among Tify-subfamilies, the Jasmonate ZIM-Domain (JAZ) family was identified as a transcriptional repressor of JA signaling [56], reinforcing the hypothesis that transcripts up-regulated by nitrate and NO-unresponsive could be related to jasmonate-signaling and resistance to biotic stress [56,57] and suggesting that the effects of nitrate nutrition on biotic stress tolerance are due to nitrate itself or to further molecules downstream its assimilation (nitrite, ammonium, or amino acids). Actually, JA and SA are well studied for their involvement in both abiotic and biotic stress responses [58,59]. Previous papers evidenced the impact of nitrogen nutrition on stress tolerance [34,60,61]. The importance of the quality of nitrogen nutrition is, therefore, relevant considering the impact of environmental stress on crop productivity. The knowledge of the underlying mechanisms and the fine-tuning management of the mineral nutrition could be helpful in defining more suitable defense practices. cluster 6 also includes transcripts with putative functions in nucleotide metabolism, such as Zm00001d049008 encoding ‘*apyrase 1′* and Zm00001d048785 encoding ‘*adenylyl-sulphate kinase 3′* (Figure 4). Apyrases (APYs) are fundamental in maintaining cellular ATP homeostasis by controlling the nucleoside triphosphate (NTP) homeostasis [62], but they are also involved in the regulation of stress adaptation [63]. Accordingly, it was shown that overexpression of *APY* could significantly inhibit ROS production and could promote stress resistance in Arabidopsis [64] and wheat [65]. The involvement of ROS homeostasis in maize root response to nitrate was already suggested by Trevisan et al. [19], but no information on the upstream signals was available. On the other hand, the adenylyl-sulphate kinase is involved in the sulphate activation for sulfonation, thus in sulfur metabolism, by catalyzing the reaction of adenosine 5’-phosphosulfate (APS) with ATP to form 3’-phosphoadenylyl-sulfate (PAPS) and ADP [66]. PAPS is then used as the universal sulfonate donor for all sulfotransferase reactions and sulfonated macromolecules are involved in many important processes, such as cell adhesion, and plant defense mechanisms [67]. These results further highlighted that the effects of nitrate nutrition on the defense response could be related to nitrate itself or to further compounds downstream its assimilation (nitrite, ammonium, or amino acids), without interplay with NO.

Finally, among genes showing an NO-independent regulation (clusters 2 and 5), the main feature for cluster 2 (transcript down-regulated by nitrate) was fucosyltransferase activity (Figure 2), protein posttranslational modification, late embryogenesis abundant (LEA) protein, and microtubule-related bins (Figure 3), while no specific transcription factors were detected for this cluster. Fucosyltransferase activity, likely related to the cell wall xyloglucan biosynthesis [68], and cytoskeleton organization are both shared with other clusters (Figure 2, Figure 3 and Figure 4). LEA protein are instead characteristic of transcript down-regulated by nitrate and NO-independent (cluster 2, Figure 3). LEA polypeptides are typically accumulated in response to water stress and act as molecular chaperones [69], but they can also be involved in the root development [70]. Terms typical of cluster 5 (up-regulated by nitrate, NO-independent) are related to abiotic stresses (heat and biodegradation of xenobiotics, Figure 3), suggesting that the resistance to heat and the detoxification from xenobiotics are independent from NO action.

As already stated, N availability affects root development in an extraordinarily complex way, by influencing its architecture at various key points and depending on many factors. The untargeted approach utilized here allowed us to identify several transcripts putatively controlling root growth and development and similarly distributed among clusters 1, 2, and 3 (Figure 6A). For six genes randomly selected among all clusters, the gene expression profiles where further confirmed by qRT-PCR (Figure 6B).

Cluster 1 included transcripts involved in root cap maturation and root protection, such as ‘*root cap-specific protein’* (Zm00001d045420), which is the homolog of Arabidopsis *GDP-D-Mannose-4,6-Dehydratase 2* (*GMD2*), and ‘*bearskin 2′* (Zm00001d003583). Clusters 2 and 3 comprised transcripts participating in root organogenesis and differential root growth (Zm00001d028803 ‘*scar2′*, Zm00001d035962 ‘*cyclops’*, Zm00001d030121 ‘*roothairless6′*, Zm00001d053163 ‘*scarecrow-like protein 26′*). Even though further research will be necessary to assess their effective role in this process, these results confirm the importance of nitrate availability in regulating the root development and further highlight the complexity of the signaling process controlling nitrate regulation of root architecture, which seems to involve both NO-dependent and NO-independent mechanisms.

The provision of cPTIO strongly inhibited the nitrate-induced lateral root development (Figure 7), leading us to definitely include NO among the signals participating in the regulation of lateral root development in response to nitrate, together with auxin and strigolactones. 

Globally, these results provide useful information on the processes and transduction signaling regulated by nitrate that are dependent on the early production of nitric oxide, further allowing us to also identify the nitrate-induced responses independent from this molecule. Many known candidate genes for root organogenesis and development were included in each cluster, highlighting the complexity of the maize root developmental response to nitrate availability. Furthermore, additional and previously unknown molecular components of the nitrate action were identified and attributed either to the NO-dependent or NO-independent responses.

Recent papers [29,30] evidenced a crucial involvement of both auxin and strigolactones, which act both in a connected and synergistic way or autonomously to regulate maize root response to nitrate. The present results suggest that the auxin signaling might at least in part depend on the NO production occurring immediately after the provision of nitrate, as also hypothesized by Manoli et al. [27]. Nonetheless, the SL action seems to directly constitute a mechanism self-directed and autonomous from NO.

## 4. Materials and Methods

### 4.1. Maize Seedlings Growth

Maize (*Zea mays* L.) B73 inbred line plants were grown as previously reported in Ravazzolo et al. [34]. In brief, seeds were rinsed in distilled water and germinated on wet filter paper at 25 °C in the dark, as described by Manoli et al. [15]. After 3 days, germinated seedlings were pre-treated for 24 h in 50 mL of an N-deprived Hoagland nutrient solution (−N) and then transferred to 3 different treatment nutrient solutions: −N (negative control), complete nutrient solution supplied with 1 mM NO_3_^−^, or complete nutrient solution supplied with 1 mM NO_3_^−^ and treated with 1 mM 2-(4-carboxyphenyl)-4,4,5,5-tetramethylimidazoline-1-oxyl-3-oxide (a NO scavenger, cPTIO, Sigma-Aldrich, Merck KGaA, Darmstadt, Germany). Seedlings were maintained in a growth chamber with a day/night cycle of 14/10 h at 25/18 °C air temperature, 70%/90% relative humidity, and 280 μmol m^−2^·s^−1^ photon flux density. For each condition, three biological replicates were analyzed (20 plants for each condition).

### 4.2. RNA Extraction and Libraries Preparation for Illumina Sequencing

Portions of 1.5 cm of root apices from the root tip cap were sampled from 20 pooled seedlings after 24 h in the treatment nutrient solution (−N, +NO_3_^−^, +NO_3_^−^ + cPTIO), in three independent biological repetitions, and immediately frozen in liquid nitrogen. Total RNA was extracted, DNAsed, and quantified as described by Ravazzolo et al. [34]. Libraries for Illumina sequencing were prepared according to the manufacturer’s instructions using the TruSeq RNA Sample Preparation kit (Illumina, San Diego CA, USA). Sequencing was performed at the Centro di Ricerca Interdipartimentale per le Biotecnologie Innovative (CRIBI, Padova, Italy), on a NextSeq500 (Illumina) instrument, producing from 23 to 35 million of 75 nt single end reads (Table S6). Datasets corresponding to −N and +NO_3_^−^ treatments were already analyzed and discussed in a previous work [34]. Here, we used them to further investigate the transcriptional regulation of root response to NO_3_^−^ provision, using cPTIO as nitric oxide (NO) scavenger to block NO signaling.

### 4.3. Processing of Sequencing Reads and Differential Expression Analysis

Base calling was performed using the Illumina Pipeline. The resulting raw reads were processed with Trimmomatic 0.33 [71] for trimming adapters and filtering low-quality reads. After quality filtering and trimming, only two biological replicates were kept for the +NO_3_^−^ + cPTIO treatment. The resulting high-quality reads were mapped to the maize B73 reference genome (RefGen ZmB73 Assembly AGPv4 and Zea_mays.AGPv4.38.gtf Ensembl Plants transcript annotation) [72] with Tophat 2.0.13 [73], as described by Ravazzolo et al. [34]. Pairwise differential expression analyses were performed with Cuffdiff v2.2.1 [74] selecting the following options: --multi-read-correct, --compatible-hits-Norm, --dispersion-method per-condition, and --library-Norm-method quartile (Supplementary Dataset S1 and S2). The genes showing a log2 fold change ratio > |1| (corresponding to a 2-fold change variation in expression level) and a false discovery rate (FDR) adjusted *p*-value ≤ 0.05 were considered as differentially expressed genes (DEGs). A Venn diagram (https://bioinfogp.cnb.csic.es/tools/venny/, accessed on 10 Febrary 2021) was used to compare and integrate DEGs responsive to nitrate and in at least one of the other treatments (−N, +NO_3_^−^ + cPTIO). Hierarchical clustering of these selected DEGs was then performed by Pearson’s correlation using the “pheatmap” package in R software (https://www.r-project.org, accessed on 14 Febrary 2021, R version 4.0.3) and displayed as a heat map of normalized, scaled, RPKM expression values. This clustering allowed us to distinguish between the NO-dependent response, the NO-independent response, and NO-modulation, each one further divided based on the up- or down-regulation on gene expression exerted by nitrate provision. RNA-Seq data from this article can be found in the Gene Expression Omnibus data library under accession number GSE173102 (https://www.ncbi.nlm.nih.gov/geo/query/acc.cgi?acc=GSE173102, accessed on 22 April 2021).

### 4.4. Gene Ontology (GO) Enrichment and Functional Annotation

GO term enrichment was determined by comparing the number of DEGs included in each cluster to the number of expressed genes in each GO term with gProfiler web-software [75]: the hypergeometric statistic for every term was used to estimate the significance of enriched pathways and processes in the gene lists and the default ontology-focused g:SCS correction method for multiple testing was applied. Maize GO annotation was retrieved from the Ensembl plant genome database. To identify the most important metabolic pathways among the six clusters, DEGs were aligned to the KEGG (Kyoto Encyclopedia of Genes and Genomes) database using the KEGG Reconstruction Pathway tool (https://www.genome.jp/kegg/, accessed on 24 Febrary 2021), and the resulting pathways were visually represents using the DiVenn tool (https://divenn.noble.org/, accessed on 25 Febrary 2021) [76]. To identify the transcription factors (TFs), the iTAK database was employed (http://itak.feilab.net/cgi-bin/itak/index.cgi, accessed on 10 Febrary 2021) [77]. The functional analysis of DEGs among the six clusters was performed using MapMan [78,79] and the overrepresentation of categories was determined using Fisher’s exact test. A critical cut-off value of 0.05 (corresponding to a Z-score ≥ 1.96) was applied to select enriched functional categories.

### 4.5. cDNA Synthesis and Quantitative Reverse Transcription PCR

The same RNA used for the RNA-seq analysis in −N and +NO_3_^−^ treatments was reverse transcribed to cDNA as described by Manoli et al. [80]. qRT-PCR was performed using the StepOne Real-Time PCR System (Applied Biosystems, Thermo Fisher Scientific, Waltham, MA, USA) as described by Nonis et al. [81]. SYBR Green reagent (Applied Biosystems, Thermo Fisher Scientific, Waltham, MA, USA) was used in the reaction, according to the manufacturer’s instructions. Melting curve analysis confirmed the absence of multiple products and primer dimers. Target gene relative expression was determined according to the Livak and Schmittgen [82] method, using *MEP* (membrane protein PB1A10.07c, Zm00001d018359) as a reference gene, according to Manoli et al. [80]. Primers were designed using the Primer3 web tool (version 4.1.0; https://primer3.ut.ee/, accessed on 23 July 2021) [83,84]. The genes and primers used are reported in Table S5.

### 4.6. Maize Seedlings Growth Analysis

Seedlings grown for 24 h in the N-deficient solution and then transferred in three different nutrient solutions for 24 h (−N, +NO_3_^−^, +NO_3_^−^ + cPTIO) were also evaluated for lateral root development phenotype.

Lateral root primordia (LRP) were visualized with a hematoxylin staining solution, and the images were analyzed with Image J Image Analysis Software as described by Ravazzolo et al. [29]. The LR density was expressed as a percentage compared to the value observed for N-deprived roots. Roots of maize seedling were separated and individually weighed. In order to obtain the average data of root weights for each tested treatment, the average weight of root was calculated for every biological repetition and then was averaged between the three biological repetitions. Three biological replicates for each treatment and an ANOVA statistic test were performed (*n* = 20).

## Data Availability

RNA-Seq data from this article can be found in the Gene Expression Omnibus data library under accession number GSE173102: https://www.ncbi.nlm.nih.gov/geo/query/acc.cgi?acc=GSE173102 (accessed on 22 April 2021).

## Supplementary Materials

The Supplementary Materials are available online at https://www.mdpi.com/article/10.3390/ijms22179527/s1.
