# Supplementary material for: Nitrate Regulates Maize Root Transcriptome through Nitric Oxide Dependent and Independent Mechanisms
Source: Int J Mol Sci. 2021 Sep 2;22(17):9527. doi: 10.3390/ijms22179527 (PMC8431222; doi:10.3390/ijms22179527)
Supplement: Supplementary file 1 [file ijms-22-09527-s001.zip › Ravazzolo_2021_Supplem-Material/Figure S1.pdf]

Nitrate regulates maize root transcriptome through nitric oxide-dependent and independent mechanisms

Laura Ravazzolo, Sara Trevisan, Silvia Iori, Cristian Forestan, Mario Malagoli, Silvia Quaggiotti*

* Department of Agronomy, Food, Natural Resources, Animal and Environment

DAFNAE, University of Padova, Viale dell'Università 16, 35020 Legnaro, Padova, Italy.

Telephone number: +39 049 8272913

E-mail: silvia.quaggiotti@unipd.it

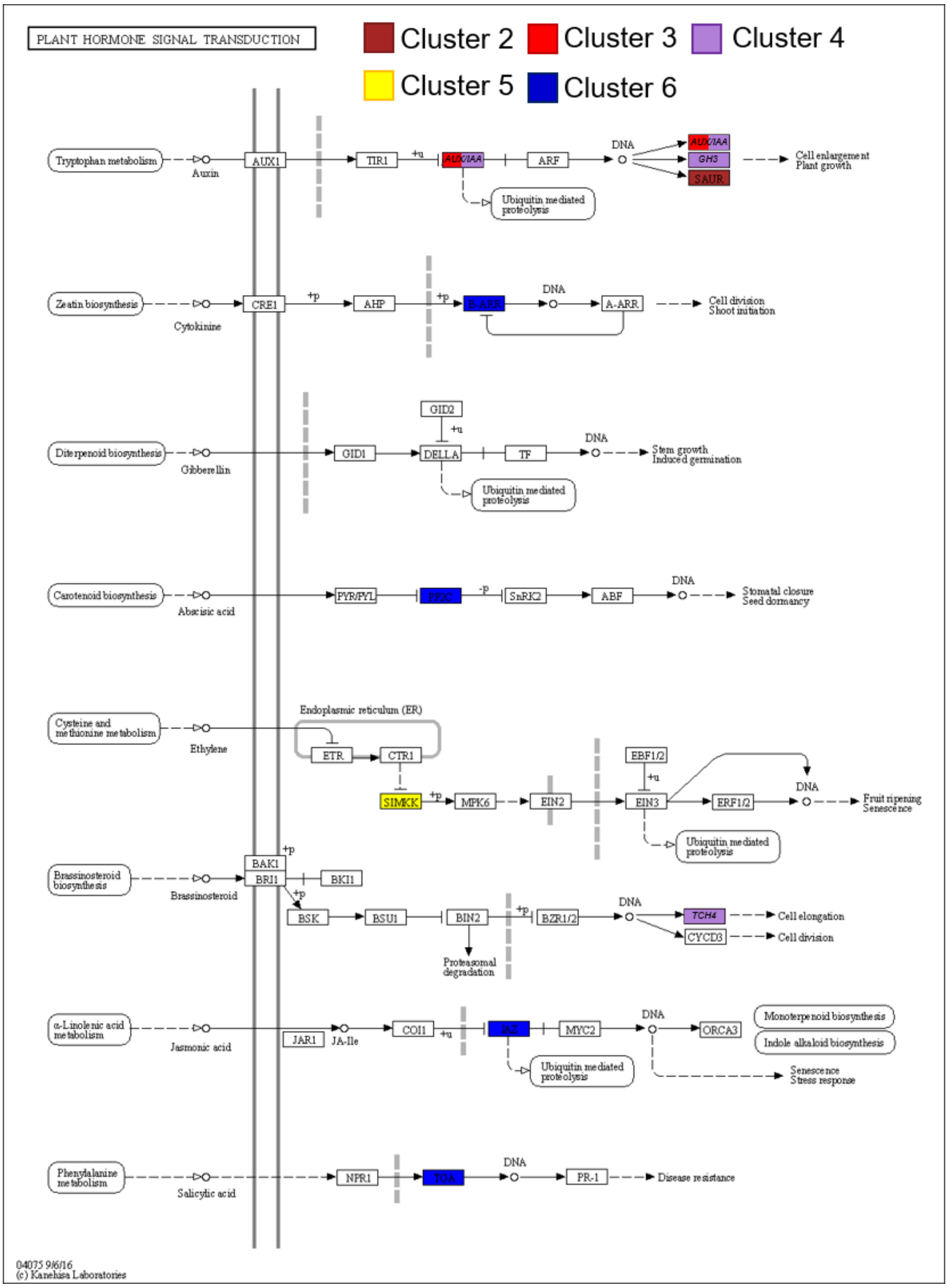

Figure S1: KEGG Search&Color Pathway representation of the pathway 'Plant hormone signal transduction' (zma04075). The analysis was set by setting cluster 2 in brown (down-regulated by nitrate, NO-independent), cluster 3 in red (down-regulated by nitrate, NO-modulated), cluster 4 in violet (up-regulated by nitrate, NO-dependent), cluster 5 in yellow (up-regulated by nitrate, NO-independent) and cluster 6 in blue (up-regulated by nitrate, NO-modulated). In this pathway, no terms were found for cluster 1 (down-regulated by nitrate, NO-dependent).
