# Supplementary material for: Nitrate Regulates Maize Root Transcriptome through Nitric Oxide Dependent and Independent Mechanisms
Source: Int J Mol Sci. 2021 Sep 2;22(17):9527. doi: 10.3390/ijms22179527 (PMC8431222; doi:10.3390/ijms22179527)
Supplement: Supplementary file 1 [file ijms-22-09527-s001.zip › Ravazzolo_2021_Supplem-Material/Suppl_Material_Captions_Ravazzolo_2021.pdf]

International Journal of Molecular Sciences

Nitrate regulates maize root transcriptome through nitric oxide dependent and independent mechanisms

Laura Ravazzolo [1], Sara Trevisan [1], Silvia Iori [2], Cristian Forestan [3], Mario Malagoli [1] and Silvia Quaggiotti [1,*]

Supplementary Materials: The following are available online at https://www.mdpi.com/article/10.3390/ijms22179527/s1:

Figure S1: KEGG Search&Color Pathway representation of the pathway 'Plant hormone signal transduction' (zma04075). The analysis was defined by setting cluster 2 in brown (down-regulated by nitrate, NO-independent), cluster 3 in red (down-regulated by nitrate, NO-modulated), cluster 4 in violet (up-regulated by nitrate, NO-dependent), cluster 5 in yellow (up-regulated by nitrate, NO-independent) and cluster 6 in blue (up-regulated by nitrate, NO-modulated). In this pathway, no terms were found for cluster 1 (down-regulated by nitrate, NO-dependent).

Table S1: Summary of gene expression values (expressed as Reads Per Kb per Million, RPKM) for all treatments analyzed (-N; +NO3-; +NO3- +cPTIO).

Table S2: Summary of gene expression values (expressed as Reads Per Kb per Million, RPKM) for DEGs in the six clusters analyzed: Cluster 1, DEGs down-regulated by nitrate provision and NO-dependent; cluster 2, DEGs down-regulated by nitrate and NO-independent; cluster 3, DEGs down-regulated by nitrate and NO-modulated; cluster 4, DEGs up-regulated by nitrate provision and NO-dependent; cluster 5, genes up-regulated by nitrate provision and NO-independent; cluster 6, DEGs up-regulated by nitrate and NO-modulated.

Table S3: Complete Gene Ontology (GO) enrichments for each of the six clusters.

Table S4: Complete KEGG Reconstruction Pathways results for each of the six clusters.

Table S5: List of genes and primers used to validate RNA-seq dataset with Real-time PCR.

Table S6: Summary statistics of RNA-Seq read sequencing, quality trimming and mapping. For each condition (-N; +NO3-; +NO3- +cPTIO) three biological replicates were processed (R1, R2, R3). Replicate R1 for the +NO3- +cPTIO treatment (highlighted in grey) did not correlate with the other replicates of the same treatment and was therefore excluded from further differential expression analysis.

Supplementary Dataset 1: Cuffdiff complete results of pairwise differential expression analyses (-N vs +NO3- +cPTIO)

Supplementary Dataset 2: Cuffdiff complete results of pairwise differential expression analyses (+NO3- vs +NO3- +cPTIO)
